# Profile and predictors of health related quality of life among type II diabetes mellitus patients in Quetta city, Pakistan

**DOI:** 10.1186/s12955-017-0717-6

**Published:** 2017-07-14

**Authors:** Qaiser Iqbal, Noman ul Haq, Sajid Bashir, Mohammad Bashaar

**Affiliations:** 1 Faculty of Pharmacy & Health Sciences, University of Baluchistan, Quetta, Pakistan; 2 Faculty of Pharmacy, University of Sargodha, Punjab, Pakistan; 3 SMART Afghan International Trainings & Consultancy, Kabul, Afghanistan

**Keywords:** HRQoL, Predictor, Profile, T2DM, Pakistan

## Abstract

**Background:**

This study aims to assess the profile and predictors of Health-Related Quality of Life (HRQoL) in Type II Diabetes Mellitus (T2DM) patients in Quetta, Pakistan.

**Methods:**

The study was designed as a questionnaire based, cross sectional analysis. 300 Type II diabetic patients attending public and private hospitals were targeted for data collection. In addition to demographic and disease related information, Euroqol Quality of Life was used to measure HRQoL. Moreover, Drug Attitude Inventory and Michigan Diabetes Knowledge Test were used to assess medication adherence and diabetes related knowledge respectively. Treatment satisfaction was assessed by patient’s experience towards health care professionals and available facilities. Descriptive statistics were used to elaborate patients’ demographic and disease related characteristics. Binary logistic regression was used to predict factors independently associated with HRQoL. SPSS v. 20 was used for data analysis and *p* < 0.05 was taken as significant.

**Results:**

Patients in the current study reported poor HRQoL with a mean score of 0.48 ± 0.36. Age, duration of disease, number of prescribed drugs, medication adherence and treatment satisfaction were significantly associated (*p* < 0.05) with HRQoL in the cross tabulation analysis. The significant variables were entered into the model that showed significant goodness of fit with highly significant Omnibus Test of Model Coefficient (Chi-square = 12.983, *p* = 0.030, df = 4). Medication adherence was reported as a significant predictor of HRQoL with an increase of one adherence score was associated with improvement of HRQoL by a factor of 1.75 provided other variables remain constant.

**Conclusion:**

The study presents a model that is associated with HRQoL with patient with T2DM, where medication adherence shaped as a predictor of HRQoL. Healthcare professionals should pay special attention on patients’ medication taking behavior and should put their efforts in explaining the benefits of the medication adherence to the patients.

## Background

Health-Related Quality of life (HRQoL) is described as an individual’s perceived quality of life, demonstrating satisfaction in the domains that are affected by health status [1]. The concept of HRQoL is frequently used in clinical research for assessing pharmaceutical care and treatment outcomes. Moreover, the literature reports HRQoL as a predictor of optimal health care service utilization [2–4]. Health-Related Quality of Life is a multidimensional construct highlighting a person’s physical, cognitive, emotional, psychological and spiritual eminence towards the current health status [5, 6]. Today, evaluation of the patients’ HRQoL is recognized as an important area of scientific knowledge, since the concept is related to the notion of health, satisfaction and well-being in the physical, psychological, socioeconomic and cultural spheres [7].

Within this context, both acute and chronic diseases are reported to adversely affect HRQoL [8]. However, in comparison to acute conditions, chronic diseases consume heavy health care resources and threaten HRQoL of the patients [9]. Shifting our concerns to the effect of Diabetes Mellitus Type II (T2DM) on HRQoL, the condition poses a serious threat to public health because of higher and long term financial and social cost involved in the management process [10]. It was reported that the number of individuals with T2DM worldwide would increase from 170 million to 370 in a span of 30 years [11–13]. Additionally, the disease is associated with both micro and macro-vascular complications with increased mortality risk from cardiac or cerebrovascular events [14]. In addition to these complications, T2DM negatively affects HRQoL as episodes and fear of hypoglycemia, change in lifestyle and fear of long-term consequences is bound to happen among patients. As the disease progresses with time, HRQoL further deteriorates with the presence of co-morbidities and multiple diabetes-related complications, consequently producing a mental, societal and financial burden on patients, caregivers and the healthcare system [15].

In a broader perspective, HRQoL is an ultimate outcome representing conclusions following a course of care. Literature does report that improved medication adherence and better disease related knowledge play a key role in improving or maintaining HRQoL in patients with chronic diseases [16]. This entails that a change in adherence or disease related knowledge occurs first, which is subsequently followed by a change in HRQoL. Therefore, we can hypothesize that patients who adhere to their treatment regimen and have better knowledge towards their disease should experience improvements in HRQoL and vice versa. Nevertheless, it is not wise to rule out other factors affecting HRQoL. For example, in the case of chronic diseases, adherence might be positively associated with side effects and perhaps lower HRQoL. On the contrary, for acute diseases, adherence to medication might be associated with a swift advancement in improving HRQoL [16].

In line with what is reported earlier, Pakistan is a developing nation with higher incidence of T2DM [17]. Despite of this alarming situation there are limited specialized diabetic centers and no formal diabetic education is provided to the patients. Very less funds are allocated to the health sector, hence health care is beyond the reach of most people in Pakistan. Moreover, 24% of the population live below the national poverty line [18] making things more complicated for patients suffering from chronic diseases. Lack of healthcare facilities, poor infrastructure and limited resources negatively affect HRQoL of population in general and of T2DM patients in particular.

Considering the rapid increase in the prevalence of T2DM in Pakistan and the unavailability of HRQoL information about the country, the aim of the study was to assess the predictors of Health Related Quality of Life in T2DM patients attending public and private health institutes of Quetta city, Pakistan. The framework was based upon the factors that are most commonly reported to affect HRQoL (i.e. Medication adherence, disease related knowledge and treatment satisfaction) [19].

## Methods

### Study design, inclusion criteria and settings

The study was designed as a questionnaire-based cross-sectional descriptive analysis. Pakistani nationals of age 18 years and above, having confirmed diagnosis of T2DM, without co-morbidities and having familiarity of Urdu (official language of Pakistan) were targeted for the study. Due to the strict inclusion criteria, it was not possible to recruit T2DM patients from one health care institute, therefore two public hospitals (Sandamen Provisional Hospital and Bolan Medical Complex Hospital) and three private hospitals (Alkahir Hospital, Alshafi Hospital and Sajid Hospital) located in Quetta city were approached for data collection.

### Sampling and inclusion criteria

A prevalence based sampling method was used to calculate the minimum sample required for this study [20]. The prevalence of T2DM in Pakistan varies from 7.6 to 11% [16, 21, 22]. Therefore, for the current study, a prevalence of 11% was taken to overcome reported variations. However, to minimize the biases in sampling, a cumulative double design was added to the sampling initial frame and 300 respondents were approached for the study [23].

n = Z^2^ × p (1 ‐ p)/d^2^

Where n = sample size, Z = confidence interval, p = prevalence of T2DM and d = margin of error.

n = 1.96^2^ × 0.11 (0.89)/0.05^2^

n = 150 × DEFF (2) (where DEFF = design effect)

n = 300

### Study variables and data collection

The first author was involved in the data collection process. In addition to the demographics, medication adherence, diabetes-related knowledge and treatment satisfaction were taken as independent variable affecting HRQoL in the regression model. The above mentioned variables were assessed via validated questionnaires and permission was taken from the developers prior to data collection.

### Assessment of medication adherence

The Drug Attitude Inventory (DAI-10) was used for the assessment of medication adherence. The instrument consists of ten items with responses in yes or no and scores ranging from 10 to −10. Patients with scores 6–10 were reported as adherent, 0–5 as moderately adherent and those with negative ranges as non-adherent [7].

### Assessment of diabetes-related knowledge

The Michigan Diabetes Knowledge Test (MDKT) was used for the assessment of diabetes-related knowledge. The MDKT was scored as zero for incorrect response and one for a correct response [24]. Therefore, the knowledge scores ranged from 0 to 14. The range of knowledge score was categorized in three different ways (poor knowledge <7, average knowledge 7–11 and good knowledge >11).

### Assessment of HRQoL

The EQ-5D is a generic HRQoL instrument developed by the EuroQoL group. It consists of five dimensions that are further divided into three levels of severity. It is a standardised instrument for use as a measure of health outcome and provides a simple descriptive profile and a single index value for health status that can be used in the clinical and economic evaluation of health care as well as population health surveys.

The EQ-5D descriptive profile consists of five dimensions (mobility, self-care, usual activities, pain/discomfort and anxiety/depression), each of which can take one of three responses. The response record three levels of severity (no problems/some or moderate problems/extreme problems) within a particular EQ-5D dimension. A total of 243 possible health states is defined in this way. Each state is referred to in terms of a 5 digit code (e.g. state 11,111 indicates no problems on any of the 5 dimensions, while state 11,223 indicates no problems with mobility and self care, some problems with performing usual activities, moderate pain or discomfort and extreme anxiety or depression). EQ-5D health states, defined by the EQ-5D descriptive system, may be converted into a single summary index by applying a formula that essentially attaches values (also called weights) to each of the levels in each dimension. The index can be calculated by deducting the appropriate weights from 1, the value for full health (i.e. state 11,111).

The visual analogue scale (VAS) is the other portion of EQ-5D consisting of a 20-cm health thermometer with two distinct end points, the best imaginable health state (score of 100) and the worst imaginable health state (score of 0). This information can be used as a quantitative measure of health outcome as judged by the individual respondents. Euroqol provided the Urdu (national language of Pakistan) version of EQ-5D upon request, and the study was registered with Euroqol [25, 26].

### Assessment of treatment satisfaction

The treatment satisfaction was based on the subjective assessment of T2DM patients’ experiences towards healthcare professionals and the available facilities. The patients were given options to state the level of satisfaction as ‘satisfied, being neutral or dissatisfied’.

### Reliability and validity of study questionnaires

The MDKT and DAI-10 are already validated in Urdu language and have proved to be a reliable tool [7, 24]. Permission to use the questionnaire was taken accordingly. EuroQol provided the validated version of EQ-5D, however a pilot study was conducted to ensure the smooth translation of the message. EQ-5D was reported as a reliable tool with an internal consistency of 0.80 with no issues related to face and content validity.

### Statistical analysis

The KS test was used for distribution analysis and non-parametric test was used accordingly. Descriptive statistics were used to describe demographic and disease characteristics of the patients. Percentages and frequencies were used for the categorical variables, while the means and standard deviations were calculated for the continuous variables. The characteristics of the whole sample, medication adherence scores, diabetes-related knowledge scores and HRQoL were presented.

Medication adherence, diabetes-related knowledge and HRQoL were calculated using the criteria originated by the developers. The association between socio-demographic data and study variables was compared through Chi square test and interpretation (Phi/Cremer’ V) was performed accordingly. The factors that were significantly associated with HRQoL were further assessed by binary logistic regression analysis. The binary logistic regression analysis included parameters with *p* value <0.05 in the Chi-square analysis. The power of independently related parameters and predictive models were expressed as odds ratio (OR) with 95% confidence intervals (CI). SPSS v. 20.0 was used for data analysis and results for all analyses were considered statistically significant at *p* < 0.05.

## Results

Three hundred T2DM patients were incorporated in the study. The description of socio-demographic variables and frequency distribution of the respondents are summarized in Table 1. The mean age (SD) of the patients was 51.25 (9.59) years, with 60.0% dominating the cohort. 92 (30.7%) had a primary level of education with 127 (42.3%) was unemployed. Nearly 55% (*n* = 166) had a family history of T2DM with 62.7% (*n* = 188) had rural residencies. 182 (60.7%) of the patients was satisfied with the present treatment and consultation.

**Table 1:** Characteristics of the study respondents (*n* = 300)

Characteristics	Frequency (*n*)	Percentage (%)
*Age group (51.25 ± 9.59)*		
30–40	43	14.3
41–50	120	40.0
51–60	90	30.0
> 60	47	15.7
*Gender*		
Male	180	60.0
Female	120	40.0
*Education*		
Primary	92	30.7
Middle	41	13.7
Metric	69	23.0
Intermediate	34	11.3
Graduate	21	7.0
Postgraduate	43	14.3
*Occupation*		
Unemployed	127	42.3
Government Employee	84	28.0
Private Employee	35	11.7
Businessman	54	18.0
*Income* ^*a*^ *(Pakistan Rupee)*		
No Income	96	32.0
< 5000	64	21.3
5000–10,000	46	15.3
10,001–15,000	13	4.3
> 15,000	81	27.0
*Locality*		
Urban	112	37.3
Rural	188	62.7
*Duration of disease (years)*		
Less than 1 year	54	18.0
1–3 years	97	32.3
3–5 years	58	19.3
More than 5 years	91	30.3
*Family history of diabetes*		
Yes	166	55.3
No	134	44.7
*Number of prescribed drugs*		
1–3	279	93.0
More than 3	21	7.0
*Overall, are you are satisfied with present treatment?*		
Satisfied	182	60.7
Neutral	33	11.0
Dissatisfied	85	28.3

^a^1 Pakistani rupee = 0.0095 US $

The HRQoL scores of respondents are presented in Table 2. The mean EQ-5D score was 0.48 ± 0.36 and the VAS score was 54.58 ± 20.28 indicating poor HRQoL in the current cohort of the patients. The patients described seventy-six different EQ-5D health states. The majority of the participants (*n* = 43, 14.3%) reported no problems/difficulties in the first, second, third and fourth domain while moderate problems/difficulties in the fifth domain (Table 3).

**Table 2:** Health related quality of life in patients with type 2 diabetes mellitus

EQ-5D Domains	Frequency (*n*)	Percentage (%)
*Mobility*		
I have no problems in walking about	217	72.3
I have some problems in walking about	61	20.3
I am confined to bed	22	7.3
*Self care*		
I have no problems with self-care	213	71.0
I have some problems washing or dressing myself	72	24.0
I am unable to wash or dress myself	15	5.0
*Usual work*		
I have no problems with self-care	148	49.3
I have some problems with performing my usual activities	118	39.3
I am unable to perform my usual activities	34	11.3
*Pain and Discomfort*		
I have no pain or discomfort	101	33.7
I have moderate pain or discomfort	164	54.7
I have extreme pain or discomfort	35	11.7
*Anxiety and depression*		
I am not anxious or depressed	37	12.3
I am moderately anxious or depressed	164	54.7
I am extremely anxious or depressed	99	33.0

Mean EQ-5D score was 0.48 ± 0.36. The VAS score was 54.58 ± 20.28

**Table 3:** Health related quality of life (Health state analysis)

Health state	Frequency	%	Health state	Frequency	%
11,111	12	4.0	22,111	1	0.3
11,112	43	14.3	22,112	2	0.7
11,113	9	3.0	22,113	1	0.3
11,121	2	0.7	22,122	2	0.7
11,122	34	11.3	22,211	1	0.3
11,123	14	4.7	21,133	2	0.7
11,131	1	0.3	21,212	2	0.7
11,132	1	0.3	21,221	2	0.7
11,133	5	1.7	21,222	4	1.3
11,212	8	2.7	21,232	1	0.3
11,213	3	1.0	21,311	1	0.3
11,221	3	1.0	21,322	3	1.0
11,222	24	8.0	21,331	1	0.3
11,223	14	4.7	22,212	1	0.3
11,232	2	0.7	22,213	1	0.3
11,233	3	1.0	22,222	9	3.0
11,312	1	0.3	22,223	4	1.3
11,313	2	0.7	22,233	2	0.7
11,322	1	0.3	22,323	5	1.7
11,323	1	0.3	22,333	1	0.3
12,113	1	0.3	23,222	1	0.3
12,121	3	1.0	23,321	2	0.7
12,122	1	0.3	23,333	1	0.3
12,123	2	0.7	31,112	1	0.3
12,213	1	0.3	31,222	1	0.3
12,222	6	2.0	32,211	2	0.7
12,223	9	3.0	32,222	1	0.3
12,233	3	1.0	32,223	1	0.3
12,321	1	0.3	32,233	4	1.3
12,332	1	0.3	32,322	1	0.3
12,333	2	0.7	32,323	1	0.3
13,212	2	0.7	32,333	2	0.7
13,222	1	0.3	33,221	1	0.3
21,111	3	1.0	33,222	1	0.3
21,112	1	0.3	33,322	1	0.3
21,121	1	0.3	33,323	1	0.3
21,122	5	1.7	33,332	1	0.3
21,123	1	0.3	33,333	3	1.0

The EQ-5D descriptive profile consists of five dimensions (mobility, self-care, usual activities, pain/discomfort and anxiety/depression), each of which can take one of three responses. The response record three levels of severity (no problems/some or moderate problems/extreme problems) within a particular EQ-5D dimension. A total of 243 possible health states is defined in this way. Each state is referred to in terms of a 5 digit code (*e.g. state 11,111 indicates no problems on any of the 5 dimensions, while state 11,223 indicates no problems with mobility and self care, some problems with performing usual activities, moderate pain or discomfort and extreme anxiety or depression*)

Table 4 presents the level of diabetes related knowledge in the current study respondents. Out of the 300 respondents, 210 (70.0%) were within the poor knowledge range, 83 (27.6%) moderate and only seven patients (2.3%) showed adequate general knowledge about T2DM. Poor knowledge was apparent in responses to questions relating to diet and disease related information. The mean knowledge score was 5.83 ± 1.92 indicating poor diabetes-related knowledge among T2DM patients.

**Table 4:** Description of diabetes-related knowledge among the study participants

Diabetes Knowledge items	Frequency (*n*)	Percent (%)	True (*n*)	False (*n*)
*The suitable diet for a diabetic is:*				
The way most Pakistani people eat	171	57.0		
A healthy diet for most people	77	25.7		
Too high in carbohydrate for most people	28	9.3	72	228
Too high in protein for most people	24	8.0		
*Which of the following is highest in carbohydrates?*				
Baked chicken	130	43.3		
Cheese	96	32.0		
Baked potato	57	19.0	54	246
Peanut butter	17	5.7		
Which of the following is highest in fat?				
Low fat milk	146	48.7		
Orange juice	117	39.0		
Corn	20	6.7	134	166
Honey	17	5.7		
*Which of the following is free food?*			27	273
Any unsweetened food	59	19.7		
Any dietetic food	187	62.3		
Any food that says sugar free on the label	26	8.7		
Any food that has less than 20 cal per serving	28	9.3		
*Glycosylated haemoglobin (Haemoglobin A1) is a test that is a measure of your average blood glucose level for the past:*				
Day	22	7.3		
Week	63	21.0		
6–10 weeks	118	39.3	131	169
6 months	97	32.3		
*Which is the best method for testing blood glucose?*				
Urine test	37	12.3		
Blood test	152	50.7	144	156
Both are equally good	111	37.0		
*What effect does unsweetened fruit juice have on blood glucose?*				
Lowers it	69	23.0		
Raises it	133	44.3	121	179
Have no effect	98	32.7		
*Which should not be used to treat low blood glucose?*				
3 hard candies	144	48.0		
½ cup orange juice	42	14.0		
1 cup diet soft drink	87	29.0	87	213
1 cup skim milk	27	9.0		
*For a person in good control, what effect does exercise have on blood glucose?*				
Raises it	220	73.3		
Lowers it	54	18.0	219	81
Has no effect	26	8.7		
*Infection is likely to cause:*				
An increase in blood glucose	114	38.0		
A decrease in blood glucose	85	28.3	83	217
Has no change on blood glucose	101	33.7		
*The best way to take care of your feet is to:*				
Look at and wash them each day	133	44.3		
Massage them with alcohol each day	66	22.0		
Soak them for 1 h each day	77	25.7	145	155
Buy shoes a size larger than usual	24	8.0		
*Eating food lower in fat decrease your risk for:*				
Nerve disease	115	38.3		
Kidney disease	60	20.0		
Heart disease	113	37.7	113	186
Eye disease	12	4.0		
*Numbness and tingling may be symptoms of:*				
Kidney disease	39	13.0		
Nerve disease	178	59.3		
Eye disease	76	25.3	181	119
Liver disease	7	2.3		
*Which of the following is usually not associated with diabetes?*				
Vision problem	23	7.7		
Kidney problem	27	9.0		
Nerve problem	30	10.0	229	71
Lung problem	220	73.3		

Knowledge was assessed by giving 1 to correct answer and 0 to the wrong answer. The scale measured knowledge from maximum 14 to minimum 0

Scores < 7 were taken as poor, 7–11 average, and > 11 good knowledge of diabetes. Mean knowledge was 5.83 ± 1.92

The DAI-10 was utilized for the assessment of adherence to medication therapy. Table 5 presents the level of adherence in the current study respondents. Among all the participants, 22 (7.3%) were low-adherers, 111 (37.0%) were medium adherers and 167 (55.6%) were considered adherent to medication therapies. The mean adherence score was 4.94 ± 2.72 indicating a moderate level of adherence in the current cohort of T2DM patients.

**Table 5:** Description of medication adherence among the study participants

Drug adherence item	False		True	
	Number	Percent	Number	Percent
For me the good things about medication outweigh the bad	177	59.0	123	41.0
I feel uncomfortable on medication	221	73.7	79	26.3
I take medications of my own choice	25	8.3	275	91.7
Medications make me more relaxed	39	13.0	261	87.0
Medication make me tired and sluggish	164	54.7	135	45.3
I take medication only when I am sick	173	57.7	127	42.3
I feel more normal on medication	17	5.7	283	94.3
It is unnatural for my mind and body to be controlled by medications	163	54.3	137	45.7
My thoughts are clearer on medication	23	7.7	277	92.3
By staying on medications, I can prevent getting sick	20	6.7	280	93.3

Adherence was assessed by giving 1 to correct answer and − 1 to the wrong answer. The scale measured adherence from a maximum of 10 to a minimum of − 10. Any negative score was rated as poor adherence, 0–5 as moderate adherence and 6–10 as good adherent. Mean adherence was 4.94 ± 2.72

Table 6 presents the cross tabulation analysis between socio-demographics and study variables. The Chi-square analysis reported a significant association between age, duration of disease, number of prescribed drugs, medication adherence and treatment satisfaction. Additionally, the association among the variables was acceptable as Cremer’ V revealed a positive and a moderate association (φc ranging from 0.55 to 0.592). No significant association was reported among other variables.

**Table 6:** Cross tabulation between demographics and study variables

Characteristics	*P*-Value
	HRQoL
Age	***0.027 (φc = 0.559)***
Gender	0.403
Education	0.060
Occupation	0.149
Income	0.413
Locality	0.895
Duration of disease (years)	***0.016 (φc = 0.564)***
Family history of diabetes	0.246
Number of prescribed drugs	***0.01 (φc = 0.592)***
Medication adherence	***0.01 (φc = 0.559)***
Diabetes-related knowledge	0.546
Treatment satisfaction	***0.037 (φc = 0.565)***

All entries in bold are significant values. The values in the brackets are the interpretation of the significant values

Logistic regression analysis of the model was performed after entering the considered independent variables and comparing it with HRQoL. In the logistic analysis age, duration of disease, number of prescribed drugs, medication adherence and treatment satisfaction was included as study parameters. The created model showed a significant goodness of fit as the Omnibus Test of Model Coefficient was highly significant (Chi square = 12.983, *p* = 0.030, DF = 4), indicating that the model was advisable. Medication adherence had significant association (adjusted OR = 1.75, 95% CI = 1.13–1.42, *P* < 0.001). Higher medication adherence scores shaped as a significant predictor of having improved HRQoL. An increase in the adherence score of one point was associated with improvement of HRQoL by a factor of 1.75 provided other factors remain constant.

## Discussion

The current study aimed to highlight the profile and predictors of HRQoL in a type II diabetic population attending public and private institutes in Quetta city, Pakistan. A number of studies report profile and predictors of HRQoL by using a model-based approach [27, 28], none is reported from Pakistan. Therefore, the current study presents a true picture of HRQoL among patients suffering from T2DM.

### Profile of HRQoL in T2DM patients

Poor HRQoL was reported among the current study respondents with a mean EQ-5D score of 0.48 ± 0.36. The utility index score was also rated as low (54.58 ± 20.28). Our findings are in agreement with what is reported by studies where T2DM was reported to negatively affect HRQoL [29, 30]. Age, duration of disease, number of prescribed drugs, medication adherence and treatment satisfaction were significantly associated with HRQoL. These findings are also comparable with the existing literature. Where Luk et al. reported a significant relationship of age with HRQoL [31], Redekop and colleagues highlighted treatment satisfaction as the only factor with a significant relationship towards HRQoL [32]. Duration of disease was also significantly associated with HRQoL and the findings are in line with what is reported by Sepulveda and colleagues from Portugal [33]. Furthermore Fincke et al. reported both number of prescribed drugs and medication adherence to be significantly associated with HRQoL that is also consistent with the current study findings [34]. However, our results were unable to present a significant association between other independent variables. A possible explanation of this difference can be attributed to the characteristics, duration and the nature of T2DM itself. Our patients were diabetic without co-morbidities and therefore had no issues with multiple therapies and medication related complications. This can be a reason contributing to the non-significant association. However, in order to accept this hypothesis, a detailed investigation on the factors cited above is warranted.

### Predictors of HRQoL

Age, duration of disease, number of prescribed drugs, medication adherence and treatment satisfaction were significant variables that were entered into the model. However, other than medication adherence non-significant association was observed between HRQoL and other independent variables. Therefore, in terms of practical implementation of the current study results, medication adherence resulted as predicting factor of HRQoL as the relationship between medication adherence and HRQoL provided significant association with highest OR value. Thus we predict an increase in overall HRQoL with an increase of medication adherence among our respondents.

In literature, the association between medication adherence and HRQoL among T2DM patients is casually described. In line to our study results, medication adherence was reported as a predicting factor towards HRQoL [35–38]. On the contrary, negative association between medication adherence and HRQoL was also reported in the literature [39, 40]. On the other hand, several studies reported no association between the two variables under discussion [41, 42]. This heterogeneity might be caused by several factors, such as differences of study designs, population, adherence measuring methods and the types of HRQoL instruments used in the respective studies.

However, while comparing our results with the studies reported from developed countries, some differences are noticed. The effect of demographic factors, such as age, sex or ethnicity, or socioeconomic factors, such as marital status, educational level, or income, on HRQoL is not specifically reported for T2DM [43]. On the other hand, macrovascular diseases and obesity [43], number of other diseases and type of treatment [44], female sex and microvascular complications [45] and to some extent high HbA1c [46] were reported as predictors of HRQoL. Such conflicting findings in the studies could be due to different factors. Different questionnaires are used for the assessment of HRQoL and studies cover different characteristics of the field of HRQoL. Additionally, it is not easy to compare results from different questionnaires because of difference of presentation and interpretation. This is the main reason why there is a marked differentiation of the results.

Within this context, adherence to therapies is a key determinant of treatment success. Failure of adherence not only affects treatment outcomes, but also leads to increase health care cost and HRQoL [47]. Much of the cost associated with non-adherence is avoidable and optimize patient adherence is one opportunity to get greater value of health care system. Improved medication adherence increases patients’ HRQoL by reducing symptoms and disease progression. A possible explanation for this relationship is explainable through the theoretical model of Self-Regulation [48]. According to the theory, it is the interest and involvement of patients in improving one’s own health that determines the success of therapy. Medication adherence is an important variable that modifies the medication taking behavior of the patients. With optimal adherence, patients can feel the difference of health status and effect of the said difference of their HRQoL. Once the desired results are attained, an overall improvement is also observed.

Diabetes Mellitus Type II is a progressive disease and pharmacological measures are needed to avoid complications and maintain glycemic control at the same time we have to remember that HRQoL is also a complex and subjective phenomena. In the broader context of pharmaceutical care there are certain other issues to be addressed. Like other developing countries, HRQoL is often neglected in Pakistan. Additionally the lack of human resources, health care facilities, income disparities and difference of living status has a profound effect on HRQoL. Even though our study findings reported medication adherence as a predictive factor of HRQoL, it is not wise to neglect other factors affecting HRQoL in the current study cohort. Therefore an in-depth study with multiple variables to assess the predictors of HRQoL in Pakistan is recommended.

#### Limitations

Like other exploratory studies, our study also has certain limitations. The data were collected from one city and cannot be generalized. Additionally, different methods of medication adherence and HRQoL assessment can reveal different result which is another limitation of the study.

## Conclusion

In conclusion, the findings of the current study presented an evidence of a model that was associated with HRQoL with patient with T2DM in Quetta, Pakistan. The current study reported medication adherence as a predictor of HRQoL. Adding to the current knowledge this is the first study that has been reported from Quetta, Pakistan. Healthcare professionals should pay special attention on patients’ medication taking behavior and should put their efforts in explaining the benefits of the medication adherence to the patients.

## Abbreviations

(CI): Confidence interval
(DAI-10): Drug attitude inventory
(HRQoL): Health related quality of life
(MDKT): Michigan diabetes knowledge test
(OR): Odds ratio
(T2DM): Type II diabetes mellitus
